# Insulin Blocks Glutamate-Induced Neurotoxicity in Differentiated SH-SY5Y Neuronal Cells

**DOI:** 10.1155/2014/674164

**Published:** 2014-06-15

**Authors:** Madhavan Nampoothiri, Neetinkumar D. Reddy, Jessy John, Nitesh Kumar, Gopalan Kutty Nampurath, Mallikarjuna Rao Chamallamudi

**Affiliations:** Department of Pharmacology, Manipal College of Pharmaceutical Sciences, Manipal University, Manipal, Karnataka 576104, India

## Abstract

Insulin is a cytokine which promotes cell growth. Recently, a few published reports on insulin in different cell lines support the antiapoptotic effect of insulin. But the reports fail to explain the role of insulin in modulating glutamate-mediated neuronal cell death through excitotoxicity. Thus, we examined the neuroprotective effect of insulin on glutamate-induced toxicity on differentiated SH-SY5Y neuronal cells. Changes in cell viability were measured by 3-(4,5-dimethylthiazol-2-yl)-2,5-diphenyl-tetrazolium bromide (MTT) based assay, while apoptotic damage was detected by acridine orange/ethidium bromide and Hoechst staining. Intracellular reactive oxygen species (ROS) accumulation and morphological alterations were also measured. Treatment with glutamate induced apoptosis, elevated ROS levels and caused damage to neurons. Insulin was able to attenuate the glutamate-induced excitotoxic damage to neuronal cells.

## 1. Introduction

Insulin is known for its action on peripheral target tissues such as liver, muscle, and adipose tissue through insulin receptors, regulating glucose uptake and utilisation, glycogen synthesis, phosphorylation or dephosphorylation of enzymes, and modulating cellular proliferation. In the brain, the presence of insulin receptor was identified years back [1, 2], but the receptor function in the CNS is still a mystery. Compared to glial cells, insulin receptors are present more in neurons [2] and are concentrated at the postsynaptic density [3]. Recent studies suggest the neurophysiological role of insulin in learning and memory [4, 5], cognition [6], and regulation of food intake [7]. The neurotrophic effects of insulin include maintenance of synaptic plasticity [8, 9] and differentiation and stimulation of neurite outgrowth [10] and circuit function [11].

Glutamate is a major excitatory neurotransmitter, widely distributed in the CNS. This excitatory aminoacid, through its action on glutamate receptors, modulates several functions of neurons including synaptic plasticity and organisation, long-term potentiation, and excitotoxicity. However, how insulin receptor signalling affects *α*-amino-3-hydroxy-5-methylisoxazole-4-propionic acid (AMPA) and* N*-methyl-D-aspartate (NMDA) receptor-mediated transmission and glutamate-mediated excitotoxicity has not been explored.

Reactive oxygen species (ROS) plays a crucial role in the pathophysiology of neurodegenerative disorders (Alzheimer's disease) and metabolic disorders (diabetes mellitus) [12, 13]. Further, ROS interacts with insulin receptor tyrosine phosphorylation, disrupting insulin signalling and affecting phosphatidyl inositol 3-kinase (PI3K) activation and insulin receptor substrate (IRS) phosphorylation [14, 15].

Insulin, after its entry into the CNS by crossing the blood brain barrier, binds to the insulin receptors located in hippocampus, cerebral cortex, cerebellum, and hypothalamus and activates intrinsic tyrosine kinase function. This triggers the subsequent signal transduction pathways. Recently, increasing body of evidence suggests the involvement of aberrant insulin receptor signalling in neurodegenerative disorders like Alzheimer's and Parkinson's disease [16, 17].

However, evidence supporting the role of insulin as a survival factor in the neuronal cells which express insulin receptors is scarce. Hence, we aimed to find out whether insulin protects neuronal cells from glutamate-induced excitotoxicity and if so whether antiapoptotic effect of insulin is mediated through oxidative stress pathway in SH-SY5Y human neuronal cells expressing insulin receptors.

## 2. Materials and Methods

### 2.1. Materials

Human recombinant insulin, acridine orange, ethidium bromide, all-trans retinoic acid (RA), dichlorofluorescein diacetate (DCFDH), 5,5′-dithiobis-(2-nitrobenzoic acid) [DTNB], Ham's F-12, Hoechst 33342, Eagle's minimum essential medium (MEM), and trypan blue were purchased from Sigma-Aldrich, USA. 3-(4,5-Dimethylthiazol-2-yl)-2,5-diphenyl-tetrazolium bromide, disodium hydrogen phosphate dihydrate, monosodium glutamate monohydrate, and fetal bovine serum were procured from HiMedia Laboratories, India.

### 2.2. Cell Culture

SH-SY5Y, neuroblastoma cell line used in this study, was obtained from the National Centre for Cell Sciences (NCCS), Pune, India. The growth medium used was 1 : 1 mixture of Eagle's minimum essential medium with nonessential amino acids and Ham's F-12 medium supplemented with 10% fetal bovine serum (FBS) and gentamicin (20 *μ*g/mL). The cells were maintained at 37°C in a CO_2_ incubator in a humidified atmosphere of 95% air and 5% CO_2_.

### 2.3. Induction of Neurotoxicity in Undifferentiated and Differentiated SH-SY5Y Cell Line Using Glutamate

Twenty four hours after seeding, 5% RA (10 *μ*M) was added to induce differentiation. Every two days, the medium was replaced with fresh medium containing RA (10 *μ*M) [18]. On day 6, to investigate the excitotoxicity of L-glutamate, both undifferentiated and differentiated cells were treated with different concentrations of L-glutamate ranging from 5 to 80 mM [19] and then 20 mM was used for further studies as it produced a significant toxicity of around 35% cell death in differentiated cells.

### 2.4. Cell Viability by MTT Assay [20]

3-(4,5-Dimethylthiazol-2-yl)-2,5-diphenyl-tetrazolium bromide (MTT) assay was used to determine cell viability. Briefly, differentiated cells in 96-well culture plate were incubated with different concentrations of insulin (0.01, 0.1, 1, and 10 *μ*M) and then exposed to 20 mM glutamate. After 48 hours of incubation, 30 *μ*L (4 mg/mL) of MTT reagent was added to each well followed by DMSO (100 *μ*L). The optical density was measured at 540 nm using a microplate reader (BioTek instruments, USA). The absorbance of the control group was considered as 100% of the cell viability. Percentage cell viability in each group was calculated.

### 2.5. Detection of Apoptotic Cells with Acridine Orange/Ethidium Bromide (AO/EB) and Hoechst Staining

The cells were stained with fluorescent DNA binding dyes AO/EB and Hoechst 33342. In brief, differentiated cells were incubated with glutamate (20 mM) alone and with different concentrations of insulin prior to glutamate exposure. After 48 hours of incubation, the medium containing drug was removed. The plate was washed and dried and 200 *μ*L of AO/EB reagent or Hoechst reagent was added to each well and incubated at 37°C for 10 minutes and observed under Nikon eclipse TS100 inverted microscope using fluorescence filters. An excitation wavelength of 460 nm and an emission maximum of 650 nm were used in AO/EB staining and, in Hoechst staining, 360 nm and 460 nm were used as excitation and emission wavelength, respectively. Three hundred cells were observed and the number of cells with apoptotic morphology appearing as condensed or fragmented nuclei was counted and expressed as percentage [21]. Apoptotic and necrotic cells were identified based on the staining pattern described by Ribble et al., 2005 [21]. Live cells were observed with normal green nuclei, early apoptotic cells with bright green nuclei with condensed or fragmented chromatin, late apoptotic cells with condensed and fragmented orange colored chromatin, and necrotic cells with structurally normal orange nuclei.

### 2.6. Intracellular Reactive Oxygen Species (ROS) Assay [22]

The differentiated cells were incubated with different concentrations of insulin (0.01, 0.1, 1, and 10 *μ*M) and then exposed to 20 mM glutamate. After 48 hours of incubation with insulin at different concentrations, the cell culture supernatants were discarded and replaced with 100 *μ*L of DCFDA (100 *μ*M). Following 1-hour incubation, the wells were washed with sterile HBSS at 37°C. HBSS (100 *μ*L) was then added to each well and the fluorescence intensity was measured using a fluorescence microplate reader at an excitation wavelength of 488 nm and emission wavelength of 525 nm. The ROS level was calculated with respect to normal control.

### 2.7. Study of Morphological Alterations and Estimation of Neurite Length

After 48 hours of incubation of differentiated SH-SY5Y cells with treatment, cells were viewed using an inverted microscope (Nikon) under 40X objective. These images were then compared to assess the effect of various treatments on the morphology of cells. For estimation of neurite length, approximately 100 images were acquired randomly from each well by scanning the wells from left to right and top to bottom. These images were then extracted to grey scale and neurite lengths were traced and measured using the public domain NIH Image J Software supplemented with Neuron J plug-in [23]. Length was defined as the straight-line distance from the tip of the neurite to the junction between the cell body and neurite base. The length (*μ*m) of neurite for each treatment was calculated.

### 2.8. Statistical Analysis

Data were statistically analysed using GraphPad Prism 5.0 software. Values are expressed as mean ± SEM of three tests in triplicate and statistical comparisons were made by one-way ANOVA followed by Tukey's multiple comparison test, where *P* < 0.05 was considered statistically significant.

## 3. Results

### 3.1. Effect of Glutamate on Cell Viability in SH-SY5Y Cells

**Figure 1:**
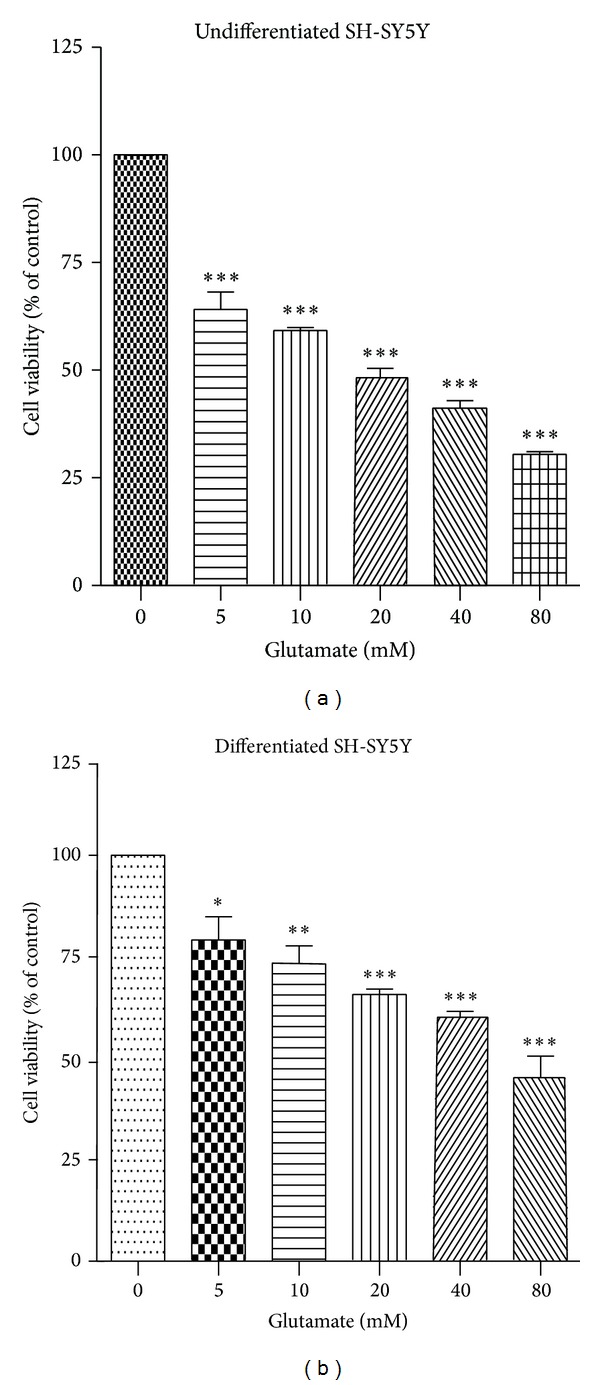
Effect of different concentrations of glutamate on cell viability in (a) undifferentiated SH-SY5Y cells and (b) differentiated SH-SY5Y cells. Values are expressed as mean ± SEM of three tests in triplicate. Statistical analysis was done by using one-way ANOVA followed by Tukey's multiple comparison test. ∗*P* < 0.05, ∗∗*P* < 0.01, and ∗∗∗*P* < 0.001 compared to control group.

In undifferentiated and differentiated cells, glutamate treatment resulted in a significant decrease in cell viability in a concentration-dependent manner (Figures 1(a) and 1(b)). After differentiation, MTT assay showed an increase in the number of viable cells compared to undifferentiated cells after exposure to glutamate, which indicates that RA differentiated cells (CTC_50_ value 70.36 mM) are less susceptible to glutamate toxicity than undifferentiated cells. Treatment with glutamate 20 mM produced about 35% cell death in differentiated cells and this concentration was used for further studies (Figure 1(b)).

### 3.2. Effect of Insulin on Glutamate-Induced Viability Loss in Differentiated SH-SY5Y Cells

Treatment with insulin increased the growth of SH-SY5Y cells compared to control cells. Maximum cell viability was observed at 1 *μ*M of insulin (Table 1). Cytotoxicity induced by glutamate treatment reduced the viability of cells to 65.80 ± 1.316% (Table 2). Insulin pretreatment at all tested concentrations (0.01 *μ*M–10 *μ*M) prevented the glutamate-induced cytotoxicity. Maximum protection was observed at 0.1 *μ*M of insulin pretreatment (Table 2).

### 3.3. Effect of Treatments on Glutamate-Induced Apoptosis in SH-SY5Y Cells

#### 3.3.1. Acridine Orange/Ethidium Bromide (AO/EB) Staining

**Figure 2:**
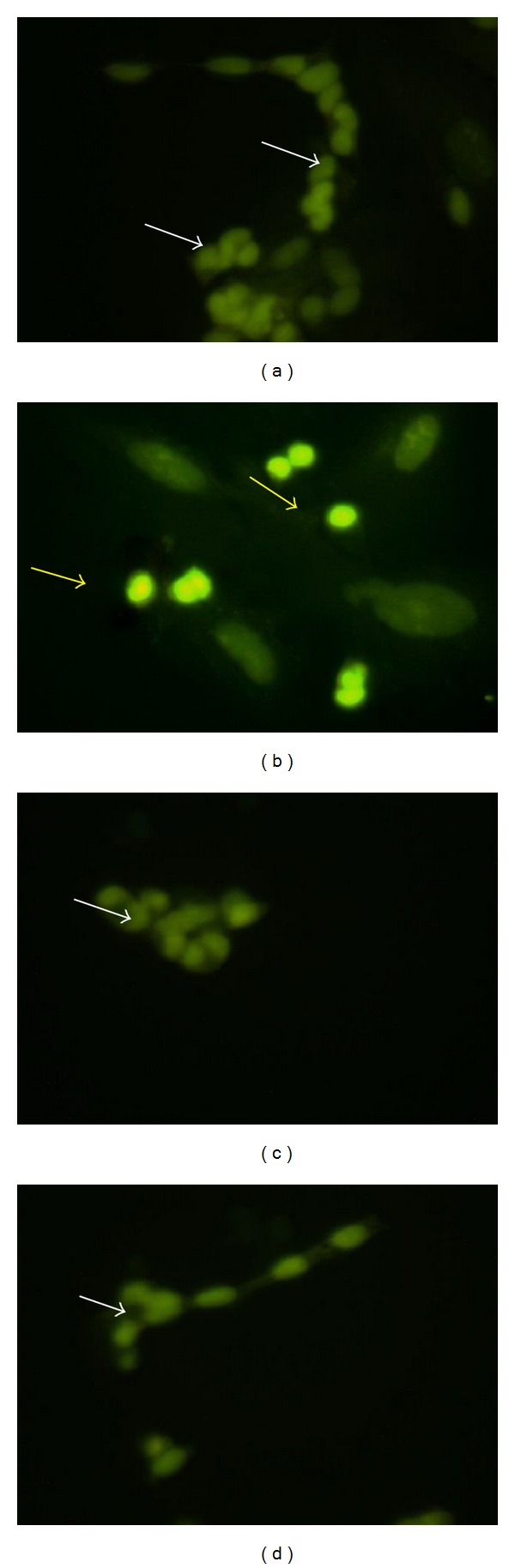
AO/EB staining in differentiated SH-SY5Y cells after treatment. (a) Control, (b) glutamate (20 mM), (c) glutamate (20 mM) + insulin (0.1 *μ*M), and (d) glutamate (20 mM) + insulin (1 *μ*M). White arrow: live cell; yellow arrow: apoptotic cell.

Control cells were found to have less than 10% of apoptotic cells (6.50 ± 2.50%). Treatment with glutamate at 20 mM concentration significantly (*P* < 0.01) increased the apoptosis (35.33 ± 2.91%) compared to control cells. Pretreatment with insulin significantly (*P* < 0.01) prevented the morphonuclear changes induced by glutamate (20 mM) in cells at both tested concentrations (0.1 *μ*M and 1 *μ*M) compared to glutamate alone (Figure 2 and Table 2).

#### 3.3.2. Hoechst 33342 Staining

**Figure 3:**
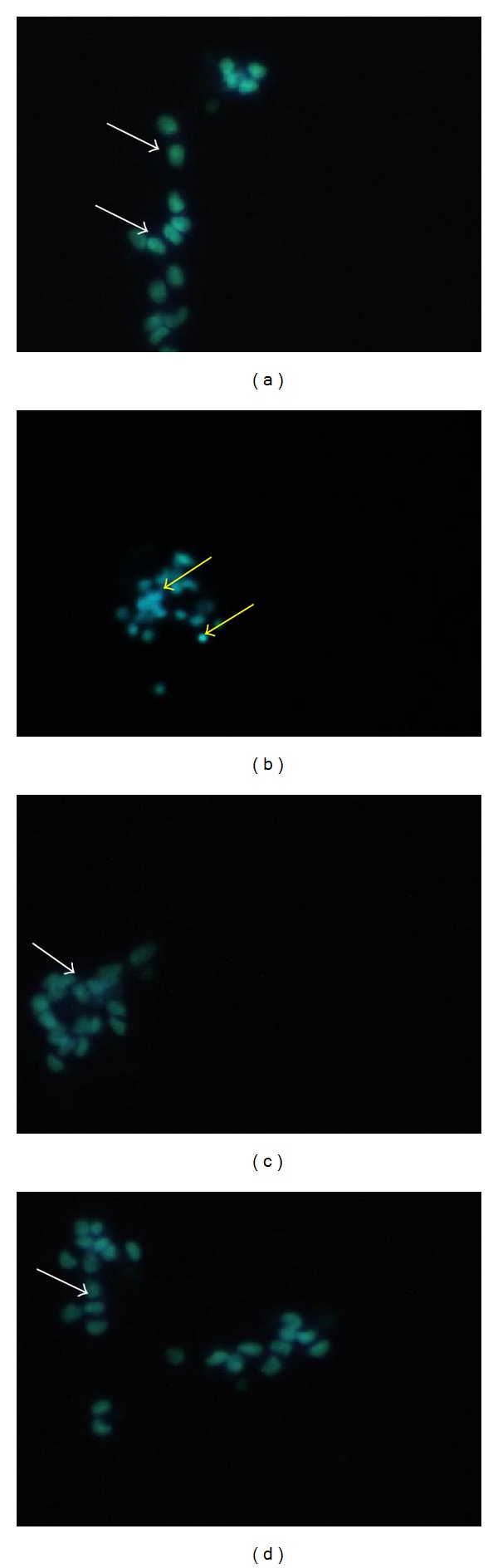
Hoechst staining in differentiated SH-SY5Y cells after treatment. (a) Control, (b) glutamate (20 mM), (c) glutamate (20 mM) + insulin (0.1 *μ*M), and (d) glutamate (20 mM) + insulin (1 *μ*M). White arrow: live cell; yellow arrow: apoptotic cell.

Hoechst 33342 staining also showed similar results to that of AO/EB staining. Control cells were found to be healthy with less than 10% of apoptotic cells (8.00 ± 2.31%). Treatment with glutamate (20 mM) increased the apoptosis (27.33 ± 2.91%) in cells significantly (*P* < 0.01) compared to control cells. Insulin pretreatment at both tested concentrations significantly prevented apoptosis induced by glutamate compared to glutamate alone (Figure 3 and Table 3).

### 3.4. Effect of Insulin on Glutamate-Induced Morphological Alterations in Neurite Length

**Figure 4:**
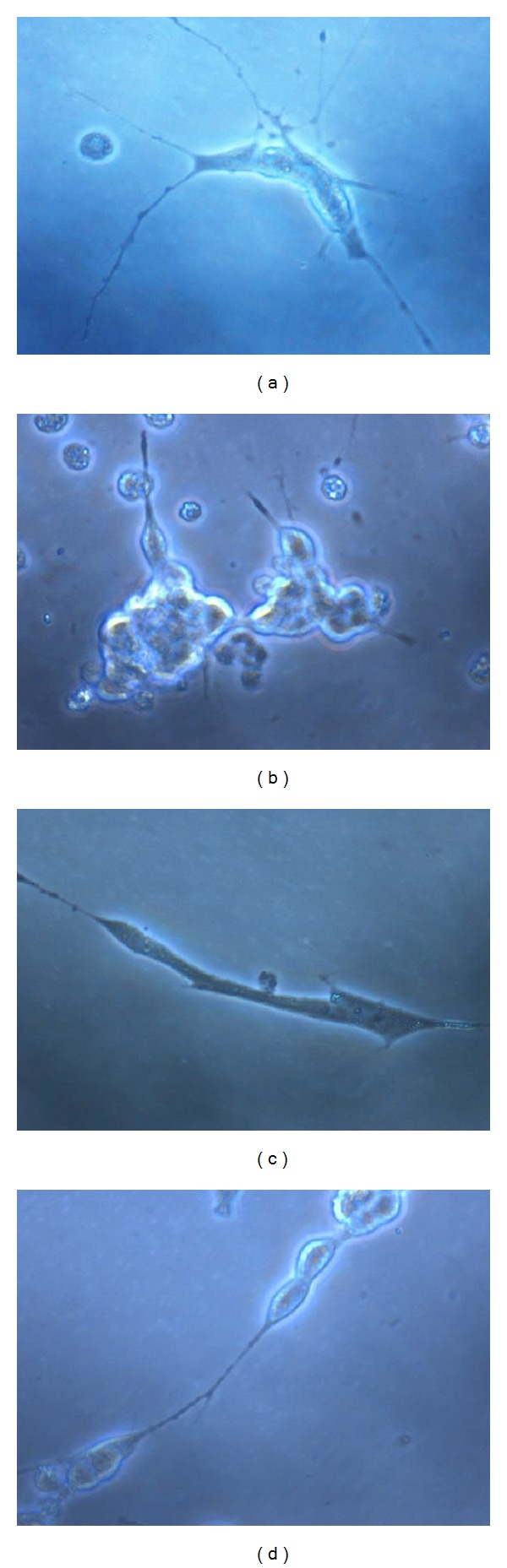
Effect of treatments on morphology of differentiated SH-SY5Y cells. (a) Control, (b) glutamate (20 mM), (c) glutamate + insulin (0.1 *μ*M), and (d) glutamate + insulin (1 *μ*M).

RA treatment caused differentiation in SH-SY5Y control cells, as evidenced by a neurite length of 799.0 ± 66.70 *μ*m. In differentiated cells, glutamate treatment significantly (*P* < 0.01) decreased the neurite length (190.1 ± 12.83 *μ*m) compared to control cells. Insulin pretreatment at both tested concentrations (0.1 *μ*M and 1 *μ*M) significantly minimised the glutamate-induced decrease in neurite length (Figure 4 and Tables 4 and 5).

### 3.5. Intracellular Reactive Oxygen Species (ROS) Assay in SH-SY5Y Cells

**Figure 5:**
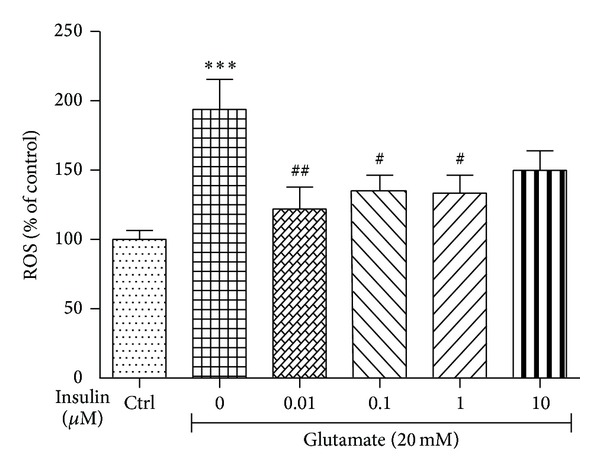
Effect of insulin pretreatment on the glutamate-induced ROS accumulation in differentiated SH-SY5Y cells. Values are expressed as mean ± SEM of three tests in triplicate. Statistical analysis was done by using one-way ANOVA followed by Tukey's multiple comparison test. ∗∗∗*P* < 0.001 compared to control group, ^#^ *P* < 0.05 compared to glutamate group, and ^##^ *P* < 0.01 compared to glutamate group.

Glutamate treatment produced a twofold increase in the ROS formation in differentiated SH-SY5Y cells. Treatments with insulin at all tested concentrations significantly minimised the glutamate-induced ROS formation in a dose-dependent manner. The maximum ROS inhibitory effect was seen at 0.01 *μ*M of insulin pretreatment (Figure 5).

## 4. Discussion

Due to sedentary lifestyle and increased life expectancy, the incidence of neurodegenerative disorders like Alzheimer's disease, Parkinson's disease, and so forth is increasing year by year. In neurodegenerative disorders, the major mechanisms responsible for neuronal cell death include excitotoxicity, oxidative stress, apoptosis, and protein (*α*-synuclein, *β*-amyloid) deposition [24–27]. In the present study, we evaluated the ability of insulin to antagonize the glutamate-induced excitotoxicity in differentiated SH-SY5Y cells. Further, we examined the antiapoptotic potential and ability of insulin to reduce oxidative stress.

Since the neuroblastoma cell lines like SH-SY5Y lack many of the features of mature neurons, differentiation is needed to induce neuron-like properties such as neurite outgrowth and morphological changes [28, 29]. In the present study, retinoic acid (10 *μ*M) was used to induce differentiation.

Glutamate has several functions to perform in the CNS as an excitatory neurotransmitter. However, it is highly toxic to neurons due to its property to cause excitotoxicity to neuronal cells. Neuronal cell death results from increased calcium load and activation of proteases and generation of ROS and nitric oxide induced by activation of glutamate receptor stimulation [30]. Several environmental toxins like kainic acid and domoic acid that act as agonists on glutamate receptor have been shown to induce neurodegenerative conditions [31, 32]. Glutamate-induced cytotoxicity has been demonstrated in various neuronal cell lines [19, 33, 34]. In the present study, glutamate exposure at various concentrations resulted in significant toxicity in both differentiated and undifferentiated cells. But after differentiation, an increase in the cell viability was observed and it correlates well with the finding that RA-induced differentiation leads to upregulation of survival signalling and reduced susceptibility to neurotoxins [18, 35]. In the present study, treatment of SH-SY5Y cells with various concentrations of insulin alone did not produce any cytotoxicity to the cells, whereas insulin at all tested concentrations significantly prevented glutamate- (20 mM) induced loss of cell viability. The maximum cell viability was observed at 0.1 *μ*M; thereafter, cell viability decreased with increase in dose, indicating the dose-dependent antagonizing effect of insulin against glutamate. Evaluation of apoptotic cells by Hoechst 33342 and AO/EB staining reveals the antiapoptotic nature of insulin in neuronal cells. Further, insulin significantly reversed glutamate-induced damage to neurons as indicated by increased neurite length. Glutamate-induced cytotoxicity also involves the generation of reactive oxygen species [36, 37]. In the present study, exposure of SH-SY5Y cells to glutamate led to elevation in the level of reactive oxygen species. Treatment with insulin reversed glutamate-induced elevation in reactive oxygen species level.

In excitatory receptor trafficking, insulin causes the internalization of AMPA receptors, resulting in long-term depression of excitatory synaptic transmission crucial for the storage of information in the brain [38, 39]. Further insulin stimulates the synthesis of PSD-95, a dendritic scaffolding protein that holds the cytoskeletal elements and receptors at synapses [40]. Modulation of NMDA-mediated synaptic transmission is also postulated [41]. Optimization of connections in brain circuits requires a mature synaptic connection at glutamatergic synapses. For this conversion of silent glutamatergic synapses into functional synapses is required [42, 43]. This is attained through redistribution of AMPA receptor [16], which in turn mediates synaptic transmission through strengthening glutamatergic synapses. But the basis of AMPA transmission mediated through insulin receptor signalling still remains controversial and needs to be studied further.

The biological significance of ROS in neurodegeneration is well documented. The intracellular calcium overload due to glutamatergic receptor stimulation and oxidative stress may lead to caspase-3 activation resulting in cell death. Insulin reduced glutamate-triggered ROS production in our study, which may be due to the ability of insulin to suppress caspase-3 activity [44]. The downstream cascades of insulin receptor, the PI3K/Akt/mTOR, and Ras/MAPK pathways, implicated for antiapoptotic action of insulin in peripheral tissues, may also play a major role in protecting SH-SY5Y cells from apoptosis due to oxidative stress in response to insulin.

## 5. Conclusion

Thus, the present study demonstrated the neuroprotective effect of insulin against glutamate-induced neurotoxicity in differentiated SH-SY5Y neuronal cells that express insulin receptor by multiple mechanisms involving reduction in glutamate-induced cell loss, prevention of apoptosis, and reducing the production of reactive oxygen species.

## Figures and Tables

**Table 1:** Effect of treatments on cell viability in differentiated SH-SY5Y cells.

Treatment	Percentage cell viability
Control	100.00 ± 0.012
Insulin (0.01 *μ*M)	110.66 ± 7.2
Insulin (0.1 *μ*M)	149.09 ± 17.0
Insulin (1 *μ*M)	165.83 ± 10.0∗
Insulin (10 *μ*M)	139.67 ± 14.3

Values are expressed as mean ± SEM of three tests in triplicate. Statistical analysis was done by using one-way ANOVA followed by Tukey's multiple comparison test. ∗*P* < 0.05 compared to control group.

**Table 2:** Effect of treatments on cell viability in differentiated SH-SY5Y cells in the presence of glutamate (20 mM).

Treatment	Percentage cell viability
Control	100.00 ± 0.012
Glutamate (20 mM)	65.80 ± 1.316∗
Glutamate + insulin (0.01 *μ*M)	108.01 ± 1.8^##^
Glutamate + insulin (0.1 *μ*M)	139.88 ± 10.4^###^
Glutamate + insulin (1 *μ*M)	132.89 ± 9.5^###^
Glutamate + insulin (10 *μ*M)	128.13 ± 5.4^###^

Values are expressed as mean ± SEM of three tests in triplicate. Statistical analysis was done by using one-way ANOVA followed by Tukey's multiple comparison test. ∗*P* < 0.05 compared to control group. ^##^ *P* < 0.01 and ^###^ *P* < 0.001 compared to glutamate alone.

**Table 3:** Percentage of apoptotic cells in differentiated SH-SY5Y cells after treatment.

Treatment	Apoptotic cells (% of total)
Control	6.50 ± 2.50
Glutamate (20 mM)	35.33 ± 2.91∗∗
Glutamate + insulin (0.1 *μ*M )	11.67 ± 3.71^##^
Glutamate + insulin (1 *μ*M)	13.22 ± 2.79^##^

Values are expressed as mean ± SEM of three tests in triplicate. Statistical analysis was done by using one-way ANOVA followed by Tukey's multiple comparison test. ∗∗*P* < 0.01 compared to control group. ^##^ *P* < 0.01 compared to glutamate group.

**Table 4:** Percentage of apoptotic cells in differentiated SH-SY5Y cells after treatment.

Treatment	Apoptotic cells (% of total)
Control	8.00 ± 2.31
Glutamate (20 mM)	27.33 ± 2.91∗∗
Glutamate + insulin (0.1 *μ*M )	13.00 ± 2.00^#^
Glutamate + insulin (1 *μ*M)	11.67 ± 2.90^#^

Values are expressed as mean ± SEM of three tests in triplicate. Statistical analysis was done by using one-way ANOVA followed by Tukey's multiple comparison test. ∗∗*P* < 0.01 compared to control group. ^#^ *P* < 0.05 compared to glutamate group.

**Table 5:** Effect of treatments on length of neurites.

Treatment	Neurite length (*μ*m)
Control	799.0 ± 66.70
Glutamate (20 mM)	190.1 ± 12.83∗∗
Glutamate + insulin (0.1 *μ*M )	640.46 ± 44.32^#^
Glutamate + insulin (1 *μ*M)	552.22 ± 22.90^##^

Values are expressed as mean ± SEM of three tests in triplicate. Statistical analysis was done by using one-way ANOVA followed by Tukey's multiple comparison test. ∗∗∗*P* < 0.001 compared to control group, ^#^ *P* < 0.05 compared to glutamate group, and ^##^ *P* < 0.01 compared to glutamate group.
